# Phenotypic and Transcriptional Changes of Pulmonary Immune Responses in Dogs Following Canine Distemper Virus Infection

**DOI:** 10.3390/ijms231710019

**Published:** 2022-09-02

**Authors:** Elisa Chludzinski, Johanna Klemens, Małgorzata Ciurkiewicz, Robert Geffers, Pauline Pöpperl, Melanie Stoff, Dai-Lun Shin, Georg Herrler, Andreas Beineke

**Affiliations:** 1 Department of Pathology, University of Veterinary Medicine Hannover, 30559 Hannover, Germany; 2 Center for Systems Neuroscience (ZSN), 30559 Hannover, Germany; 3 Genome Analytics, Helmholtz Centre for Infection Research, 38124 Braunschweig, Germany; 4 Institute of Virology, University of Veterinary Medicine Hannover, 30559 Hannover, Germany

**Keywords:** apoptosis, bulk RNA sequencing, canine distemper, cytokines, immunohistochemistry, innate immunity, morbillivirus, tumor necrosis factor alpha, type I interferon, viral pneumonia

## Abstract

Canine distemper virus (CDV), a morbillivirus within the family *Paramyxoviridae*, is a highly contagious infectious agent causing a multisystemic, devastating disease in a broad range of host species, characterized by severe immunosuppression, encephalitis and pneumonia. The present study aimed at investigating pulmonary immune responses of CDV-infected dogs in situ using immunohistochemistry and whole transcriptome analyses by bulk RNA sequencing. Spatiotemporal analysis of phenotypic changes revealed pulmonary immune responses primarily driven by MHC-II^+^, Iba-1^+^ and CD204^+^ innate immune cells during acute and subacute infection phases, which paralleled pathologic lesion development and coincided with high viral loads in CDV-infected lungs. CD20^+^ B cell numbers initially declined, followed by lymphoid repopulation in the advanced disease phase. Transcriptome analysis demonstrated an increased expression of transcripts related to innate immunity, antiviral defense mechanisms, type I interferon responses and regulation of cell death in the lung of CDV-infected dogs. Molecular analyses also revealed disturbed cytokine responses with a pro-inflammatory M1 macrophage polarization and impaired mucociliary defense in CDV-infected lungs. The exploratory study provides detailed data on CDV-related pulmonary immune responses, expanding the list of immunologic parameters potentially leading to viral elimination and virus-induced pulmonary immunopathology in canine distemper.

## 1. Introduction

Morbilliviruses are highly contagious pathogens that cause outbreaks of systemic, often fatal disease in animals and humans worldwide. They include measles virus (MeV), rinderpest virus (RPV), peste-des-petits-ruminants virus (PPRV), cetacean morbillivirus (CeMV), phocine distemper virus (PDV), porcine morbillivirus (PoMV) and canine distemper virus (CDV). Common features of morbillivirus infections are virus transmission by the respiratory route, transient severe immunosuppression, which favors opportunistic infections, and induction of life-long immunity in survivors [1,2]. Canine distemper, caused by CDV, is a highly prevalent infectious disease in a wide host spectrum comprising dogs, wild canids, mustelids, raccoons, bears, large felids and non-human primates [3,4,5,6,7]. It exhibits the second highest fatality rate of infectious diseases in dogs, after rabies, and may contribute to the extinction of endangered wildlife species [6,8,9,10,11,12]. Although canine distemper is controlled by vaccines in domestic dogs and farmed mink, its global distribution, promiscuity and reservoir in wildlife populations make an eradication unfeasible. To date, only supportive treatment is available for infected animals [5,13,14,15].

CDV shares many molecular and pathogenic similarities with MeV, containing a non-segmented, negative single-stranded RNA genome encoding for the nucleocapsid protein (NP), phosphoprotein (P), RNA polymerase (L), matrix protein (M) and the two surface glycoproteins fusion protein (F) and hemagglutinin (H), used for attachment, entry and exit from the host cell [16,17,18]. Following oronasal infection, morbilliviruses primarily infect alveolar macrophages and dendritic cells via the signaling lymphocyte activation molecule (SLAM) [19,20,21,22]. These cells bypass the respiratory epithelium and subsequently drain to regional lymphoid tissues for further virus replication in B and T cells [22,23,24]. During a first viremia phase, CDV disseminates to distant lymphoid tissues [22,24,25,26,27,28]. Following a second viremic phase, the virus is found in mucosal tissues, endothelial cells and the central nervous system (CNS) leading to catarrhal gastroenteritis, demyelinating encephalitis and interstitial pneumonia. CDV uses Nectin-4, an adherens junction protein expressed on epithelial cells, to enter mucosal surfaces of the respiratory, gastrointestinal and urinary tract [4,24,29,30,31]. Similar to human measles, CDV infection of the lung is characterized by bronchiolar necrosis, type II pneumocyte hyperplasia, syncytia formation and eosinophilic inclusion bodies within epithelial and immune cells [32,33,34]. From the respiratory tract, CDV is expelled to other hosts via coughing or sneezing, representing the major route of virus transmission [6,35]. The clinical course following infection is influenced by the virulence of the CDV strain, as well as the age and immune status of the host [6,36,37,38,39]. For instance, puppies and immunocompromised dogs with low antibody titers are most prone to develop fatal generalized acute distemper [6,35,37,38,39]. Older individuals with an intermediate humoral response may display persistent infection resulting in chronic progressive neurological disease [6,37,38,40].

The immune system provides several defense mechanisms against infections, which on one hand contribute to elimination of the infectious agent but may also cause harmful effects by immune-mediated tissue damage [41,42,43]. For instance, pro-inflammatory cytokines, such as tumor necrosis factor (TNF)-α not only enhance protective adaptive immunity but also lead to host cell lysis and exuberant inflammation [44,45,46]. In the respiratory tract, epithelial cells form a physical barrier, which includes the mucociliary clearance mechanism and adherence through tight junctions preventing pathogen invasion [47,48]. Innate immune cells, such as alveolar macrophages, dendritic cells and natural killer cells, detect viral structures via pattern recognition receptors that results in the initiation of antiviral mechanisms, antigen presentation and virus-specific adaptive immunity [49]. Here, type I interferons trigger the synthesis of a variety of antiviral effector proteins, such as interferon-induced GTP-binding protein Mx1, protein kinase R (PKR), interferon-stimulated gene 15 (ISG15) and RNAse L, which impair viral replication and establish an anti-viral state within host cells [50].

Immune responses to CDV infection have been studied in the CNS and lymphoid organs of dogs. During acute infection, CDV elicits a dominant pro-inflammatory environment in the brain, characterized by the activation of resident immune cells, major histocompatibility complex class II (MHC-II) upregulation and transcription of interleukin (IL)-6, IL-8 and TNF-α as well as of interferon-related genes [41,51,52,53,54,55]. Similarly, an upregulation of TNF-α and depletion of Foxp3^+^ regulatory T cells can be detected in the spleen of acutely infected dogs, indicative of disturbed immune regulation [56]. In advanced phases of canine distemper, CD4^+^ T cell-mediated delayed type hypersensitivity results in immune-mediated CNS damage [52,57,58]. MeV infection of children causes depletion of T and B cells in pulmonary alveoli and bronchus-associated lymphoid tissue (BALT) of the lung, not affecting CD8^+^ T cells. It is associated with an increased IL-1, IL-4 and interferon expression and apoptosis of dendritic cells, CD4^+^ cells and natural killer cells in the lung [59]. In CeMV-infected dolphins, macrophage and apoptotic cell numbers are increased in lung tissue [60]. So far, detailed information on immune responses in the lung of CDV-infected animals is sparse.

Given the seriousness of diseases caused by morbilliviruses, it is critical to understand the mechanisms involved in the pathogenesis and immune response in the respiratory system. Therefore, the aims of the present study are: (i) to characterize spatiotemporal phenotypic changes of immune cell compositions in lungs of CDV-infected dogs in situ and (ii) to analyze pulmonary immune responses in canine distemper by means of bulk RNA sequencing.

## 2. Results

### 2.1. Histopathologic Changes in Canine Distemper Virus-Infected Lungs

**Figure 1:**
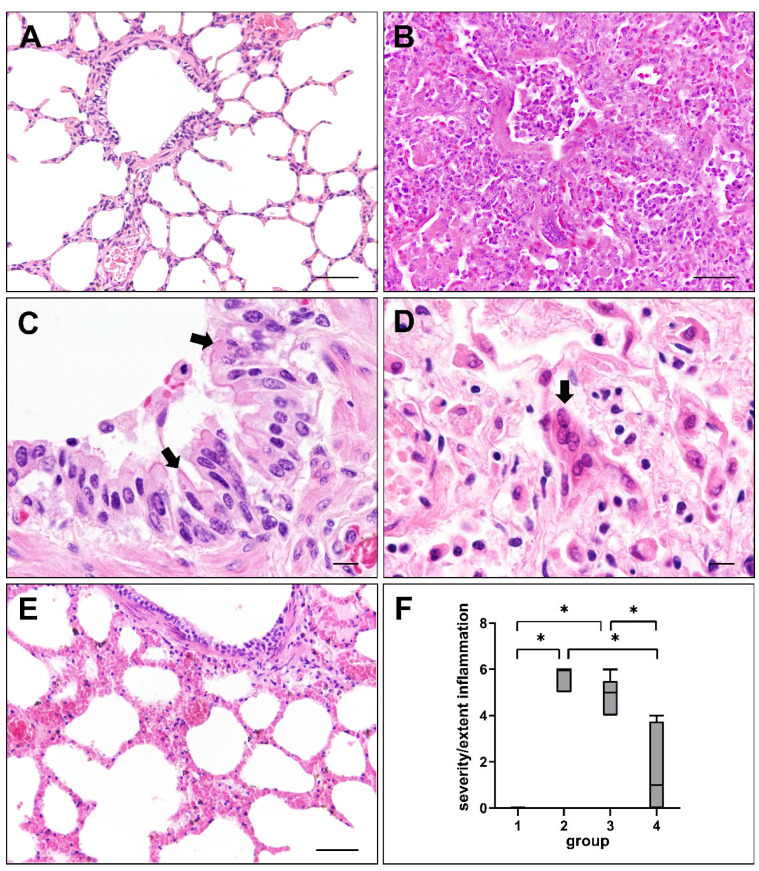
Lung lesions in canine distemper virus (CDV)-infected dogs. (**A**) Non-infected control lung. (**B**) Interstitial pneumonia of a CDV-infected dog (group 2). (**C**) Cytoplasmic viral inclusion bodies in airway epithelial cells (arrows) of a CDV-infected dog (group 3). (**D**) Syncytia formation (arrow) in a CDV-infected lung (group 2). (**E**) Mild pulmonary changes during subacute-chronic infection (group 4). (**F**) Intensity of inflammatory responses at different phases of CDV infection. Groups: 1 = non-infected controls; 2 = acute CDV infection; 3 = subacute CDV infection; 4 = subacute-chronic CDV infection; box and whisker plots display median and quartiles with maximum and minimum values. Significant differences (*p* ≤ 0.05, Kruskal–Wallis *H* test) are labelled by asterisks. Scale bars: (**A**,**B,E**): 50 µm; (**C**,**D**): 10 µm.

Lung sections were assessed for histopathological changes at different phases of CDV infection. Non-infected control dogs (group 1) did not show any significant pulmonary alterations (Figure 1A). Dogs in the acute (group 2) and subacute (group 3) phase of CDV infection exhibited interstitial pneumonia and necrotizing bronchiolitis, characterized by mononuclear cell infiltration, type II pneumocyte hypertrophy and hyperplasia, fibrin extravasation and edema. Bronchiolar epithelium showed desquamation, partially leading to complete denudation of bronchioli. Debris, sloughed epithelial cells and foamy macrophages were found within alveolar and bronchiolar lumina (Figure 1B). Cytoplasmic and/or intranuclear viral inclusion bodies were present in epithelial and inflammatory cells (Figure 1C) in 72.7% (8/11) of group 2, 77.8% (7/9) of group 3 and 12.5% (1/8) of group 4 dogs. Syncytia formation (Figure 1D) was observed in 90.9% (10/11) of group 2, 88.9% (8/9) of group 3 and 12.5% (1/8) of group 4 animals. The severity and extent of inflammatory responses, assessed by a semi-quantitative score, was significantly reduced in lungs of dogs with subacute-chronic CDV infection (group 4) compared to group 2 (*p* = 0.001) and group 3 (*p* = 0.017) dogs (Figure 1E,F).

### 2.2. Virus Loads and Cell Tropism

**Figure 2:**
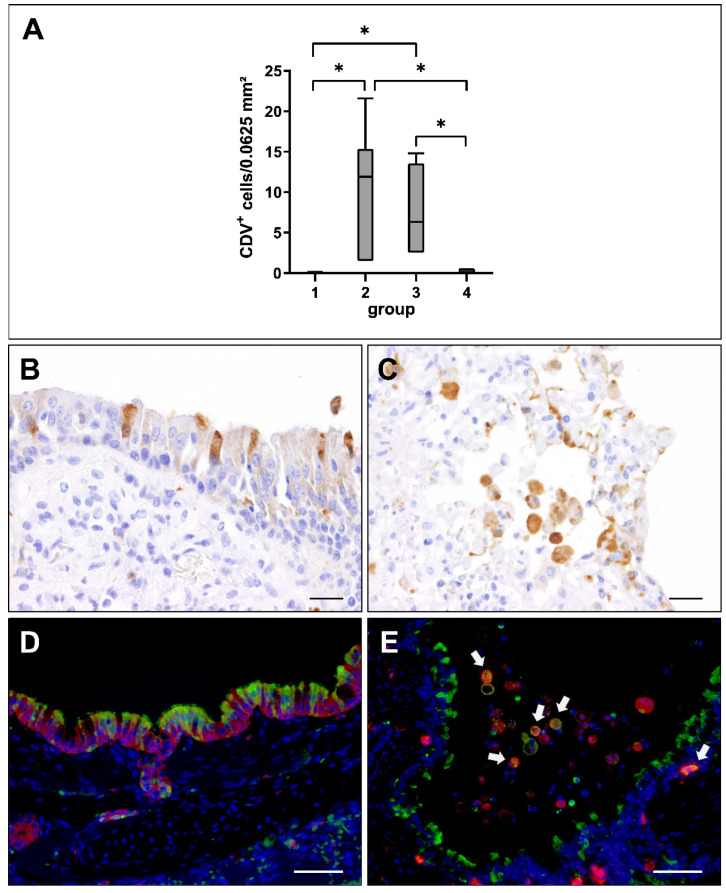
Canine distemper virus (CDV) loads and distribution in the lung. (**A**) Quantification of CDV antigen at different phases of infection. Note significant increase of CDV loads in the acute (group 2) and subacute infection phase (group 3), followed by a decrease in the subacute-chronic phase (group 4). Groups: 1 = non-infected controls; 2 = acute CDV infection; 3 = subacute CDV infection; 4 = subacute-chronic CDV infection; box and whisker plots display median and quartiles with maximum and minimum values. Significant differences (*p* ≤ 0.05, Kruskal–Wallis *H* test) are labelled by asterisks. (**B**,**C**) Immunohistochemistry revealed CDV antigen within airway epithelial cells (**B**) and intraalveolar cells (**C**). (**D**,**E**) Detection of CDV antigen (green) and cytokeratin (epithelial cells, red, **D**) and Iba-1 (histiocytic cells, red; CDV^+^ histiocytes, white arrows, **E**) by immunofluorescence. Nuclei were counterstained with bisbenzimide (blue). Scale bars: (**B**,**C**): 20 µm. (**D**,**E**): 50 µm.

Viral loads in lung tissue at different stages of CDV infection were evaluated by immunohistochemistry. CDV nucleoprotein was absent in non-infected control dogs (group 1). Lung tissue from affected dogs showed highest numbers of CDV-infected cells in the acute (group 2) and subacute stage (group 3), while CDV was almost eliminated from lungs in the subacute-chronic phase (group 4; Figure 2A). CDV loads correlated positively with the severity and extent of interstitial pneumonia (correlation coefficient: 0.658).

Immunohistochemistry revealed a preferential infection of the epithelium and of immune cells in the bronchiolar submucosa and alveolar area (Figure 2B,C). Immunofluorescence double-labeling confirmed CDV infection of cytokeratin^+^ epithelial cells as well as of submucosal and alveolar Iba-1^+^ histiocytes (Figure 2D,E).

### 2.3. Phenotyping of Pulmonary Immune Responses

Spatiotemporal changes of cellular immune responses of CDV-infected lungs were determined by immunohistochemistry in bronchial, bronchiolar and alveolar regions at different disease phases.

#### 2.3.1. Innate Immune Cell Response

**Figure 3:**
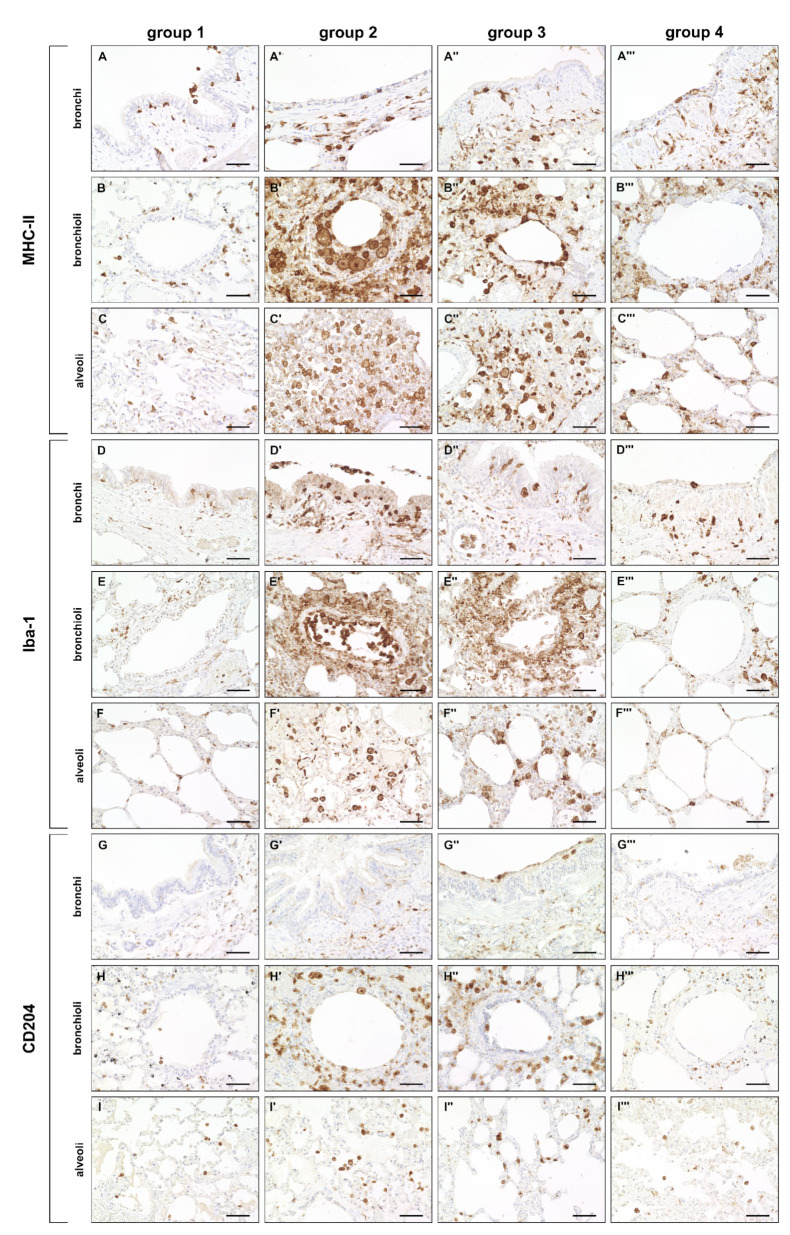
Innate cellular immune responses in canine distemper virus (CDV)-infected lungs. (**A**–**C’’’**) Major histocompatibility complex (MHC)-II^+^ cells (antigen-presenting cells) in the bronchial (**A**–**A’’’**), bronchiolar (**B**–**B’’’**) and alveolar region (**C**–**C’’’**). (**D**–**F’’’**) Ionized calcium–binding adapter molecule 1 (Iba-1)^+^ cells (macrophages) in the bronchial (**D**–**D’’’**), bronchiolar (**E**–**E’’’**) and alveolar region (**F**–**F’’’**). (**G**–**I’’’**) CD204^+^ cells (macrophages) in the bronchial (**G**–**G’’’**), bronchiolar (**H**–**H’’’**) and alveolar region (**I**–**I’’’**). Note increased numbers of MHC-II^+^ cells, Iba-1^+^ cells and CD204^+^ cells in all anatomical compartments mainly in groups 2 and 3. Groups: 1 = non–infected control lungs; 2 = acute CDV infection; 3 = subacute CDV infection; 4 = subacute-chronic CDV infection. Scale bars: 50 µm.

**Figure 4:**
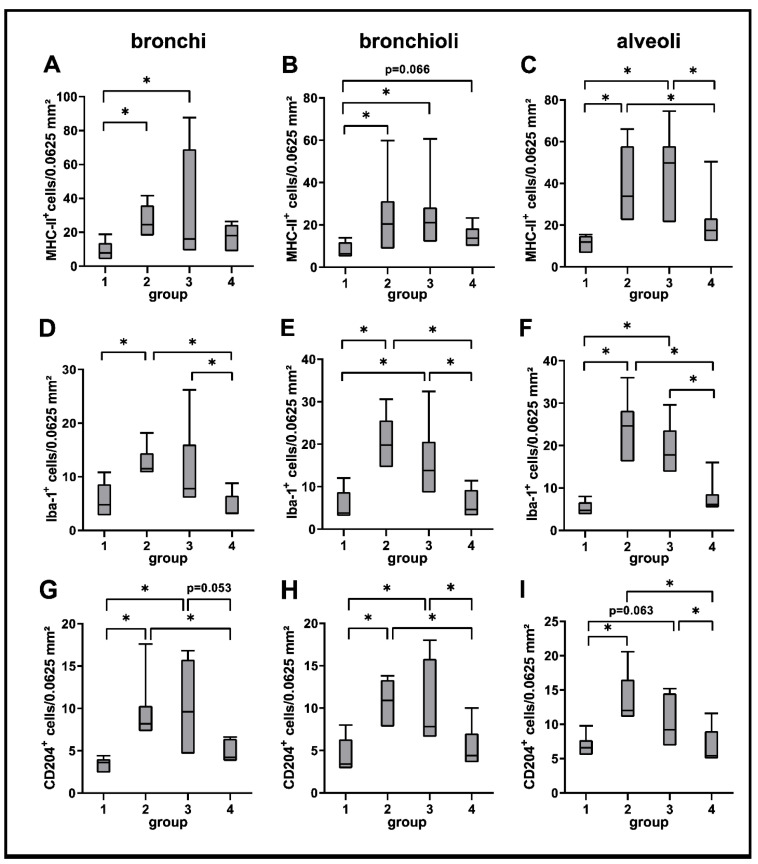
Quantification of innate cellular immune responses in canine distemper virus (CDV)-infected lungs. MHC-II (**A**–**C**), Iba-1 (**D**–**F**) and CD204 (**G**–**I**) antigen detection. Groups: 1 = non-infected control lungs; 2 = acute CDV infection; 3 = subacute CDV infection; 4 = subacute-chronic CDV infection; box and whisker plots display median and quartiles with maximum and minimum values. Significant differences (*p* ≤ 0.05, Kruskal–Wallis *H* test) are labelled by asterisks.

A significantly increased expression of MHC-II was found in bronchial, bronchiolar and alveolar regions during the acute (group 2) and subacute phase (group 3) compared to non-infected lungs (group 1), followed by a significant decrease in the subacute-chronic phase (group 4). Similarly, the number of Iba-1^+^ and CD204^+^ macrophages was significantly elevated in all three anatomical compartments in group 2 and 3 animals, and significantly decreased in lungs of group 4 animals (Figure 3 and Figure 4).

#### 2.3.2. Adaptive Immune Cell Response

**Figure 5:**
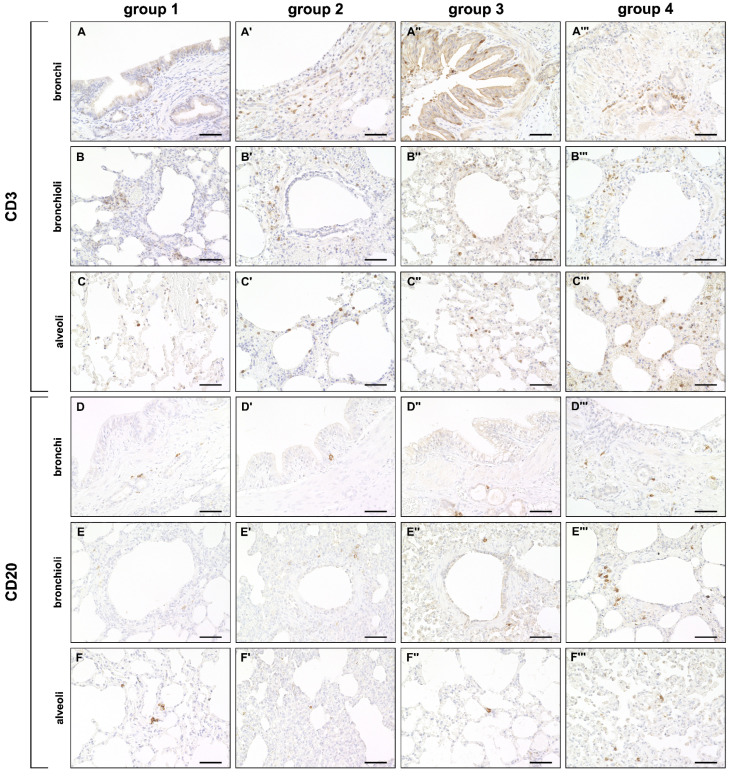
Adaptive cellular immune responses in canine distemper virus (CDV)-infected lungs. (**A**–**C’’’**) CD3^+^ cell (T cell) detection in the bronchial (**A**–**A’’’**), bronchiolar (**B**–**B’’’**) and alveolar region (**C**–**C’’’**). (**D**–**F’’’**) CD20^+^ cell (B cell) detection in the bronchial (**D**–**D’’’**), bronchiolar (**E**–**E’’’**) and alveolar region (**F**–**F’’’**). Groups: 1 = non-infected control lung; 2 = acute CDV infection; 3 = subacute CDV infection; 4 = subacute-chronic CDV infection. Scale bars: 50 µm.

**Figure 6:**
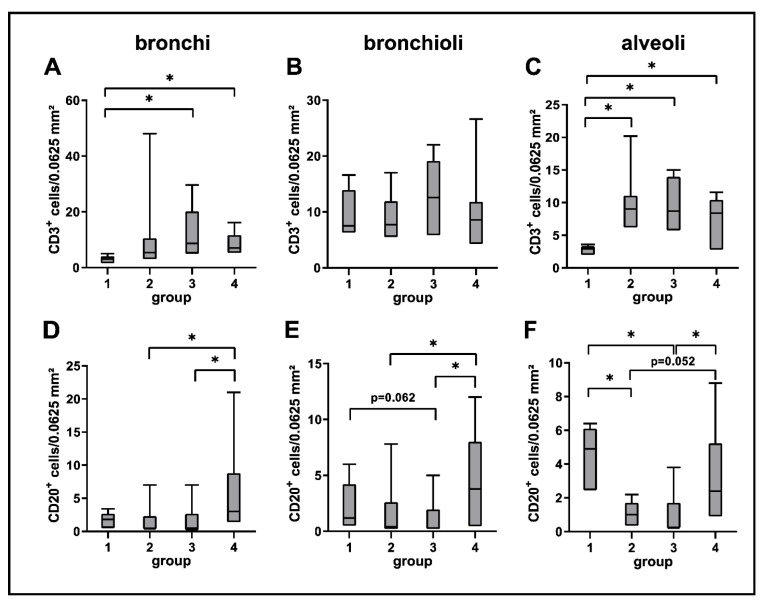
Quantification of adaptive cellular immune responses in canine distemper virus (CDV)-infected lungs. CD3 (**A**–**C**) and CD20 (**D**–**F**) antigen detection. Groups: 1 = non-infected control lungs; 2 = acute CDV infection; 3 = subacute CDV infection; 4 = subacute-chronic CDV infection; box and whisker plots display median and quartiles with maximum and minimum values. Significant differences (*p* ≤ 0.05, Kruskal–Wallis *H* test) are labelled by asterisks.

A mild but significant increase of CD3^+^ T cells was observed in bronchial and alveolar regions at all disease stages compared to control dogs. CD20^+^ B cells were decreased in alveolar regions during the acute (group 2) and subacute phase (group 3) compared to control lungs (group 1). A significant increase of CD20^+^ B cells was found during the subacute-chronic phase (group 4) around bronchi and bronchioles compared to group 2 and group 3 and in alveolar areas compared to group 3 (Figure 5 and Figure 6).

### 2.4. Global Transcriptome Analysis of Canine Distemper Virus-Infected Lung Tissue

**Figure 7:**
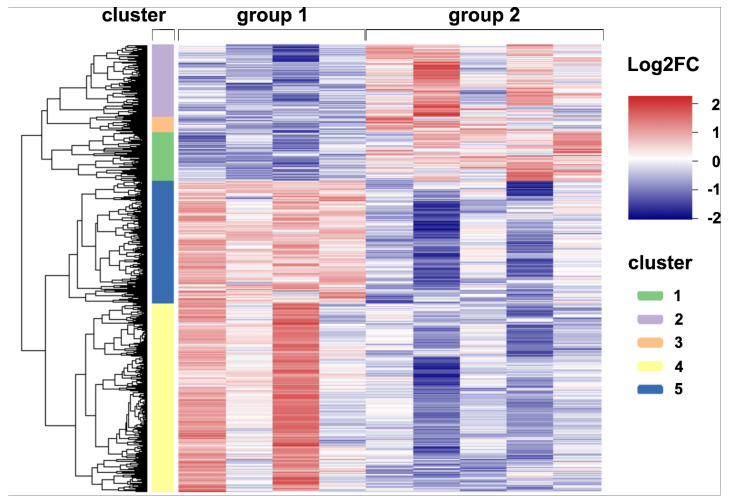
Global gene expression analyses. A heatmap illustrating the differences in expression of 3252 differentially expressed genes (DEGs) detected by pairwise comparisons of lung tissue of uninfected and CDV-infected dogs (*p* < 0.05). The color scheme demonstrates normalized gene expression z-score values ranging from −2 (relative low expression, in blue) to 2 (relative high expression, in red). DEGs were subdivided into five clusters with distinct expression profiles: Clusters 1–3 (green, purple and orange bar) contain upregulated genes (Log2FC > 0), whereas clusters 4 and 5 (yellow and blue bar) show downregulated genes (Log2FC < 0) in CDV-infected dogs. Groups: 1 = uninfected control lung; 2 = acute CDV infection.

In order to gain more detailed insights into antiviral immune mechanisms, pulmonary inflammation and responses to virus-induced injury, bulk RNA-Seq transcriptome analysis of lung tissue from non-infected and acutely CDV-infected dogs was performed. In total, 3252 differentially expressed genes (DEGs, *p* < 0.05) were detected by direct comparison between non-infected control dogs and CDV-infected dogs (Supplementary Table S1). Hierarchical cluster analysis resulted in five clusters, grouping genes with similar expression patterns. Genes in cluster 1 (n = 353), cluster 2 (n = 515) and cluster 3 (n = 118) were upregulated in CDV-infected dogs, whereas clusters 4 (n = 1373) and 5 (n = 893) contained downregulated genes (Figure 7).

Enrichment analysis using gene ontology (GO) terms in the category “biological function”, showed an overrepresentation of terms such as “defense response to virus”, “negative regulation of viral process”, “response to type I interferon”, “mononuclear cell proliferation”, “cytokine-mediated signaling pathway” and “regulation of apoptotic process” in cluster 1 and 2. No GO terms were enriched in cluster 3 (Table 1, for details see Supplementary Table S2).

Clusters 1 and 2 contained DEGs encoding for several pro-inflammatory cytokines, which were significantly upregulated in CDV-infected lungs. These included genes encoding for IL-6 (*p* = 0.013), IL-12 (*p* = 0.001) and TNF-α (*p* = 0.005). Counter-regulatory cytokines, such as IL-10 and transforming growth factor beta (TGF-β), showed no significant changes in their transcription rate when compared to control dogs. Moreover, a significantly increased expression of genes related to positive regulators of apoptosis, such as programmed death ligand 1 (PDL-1, *p* = 0.001), promyelocytic leukemia protein (PML, *p* = 0.003) and various caspases, such as caspase-8 (*p* = 0.017) and caspase-12 (*p* = 0.005) was observed in CDV-infected dogs. Transcripts with the highest upregulation compared to control dogs included many interferon-induced genes such as *MX1* (encoding for Mx1, *p* = 0.001), eukaryotic translation initiation factor 2 alpha kinase 2 (*EIF2AK2,* encoding for PKR, *p* = 0.001), *ISG15* (*p* = 0.001), 59 kDa 2’-5’-oligoadenylate synthetase-like protein (*OASL*, *p* = 0.001), 2’-5’-oligoadenylate synthetase 1 (*OAS1*, *p* = 0.001) and *RNASEL* (encoding for RNAse L, *p* = 0.003).

Enrichment analysis of clusters 4 and 5, containing genes downregulated in CDV-infected dogs revealed terms such as “cilium assembly”, “cilium movement” and “epithelial cell proliferation”. Downregulated genes included dynein axonemal intermediate chain 3 (*DNAI3*), HOATZ cilia and flagella associated protein (*HOATZ*), cilia and flagella associated protein 221 (*CFAP221*) and growth differentiation factor 5 (*GDF5*), indicative of ciliary dysfunction and impaired tissue repair (Supplementary Tables S1 and S2).

### 2.5. Cytokine Expression Analyses

**Figure 8:**
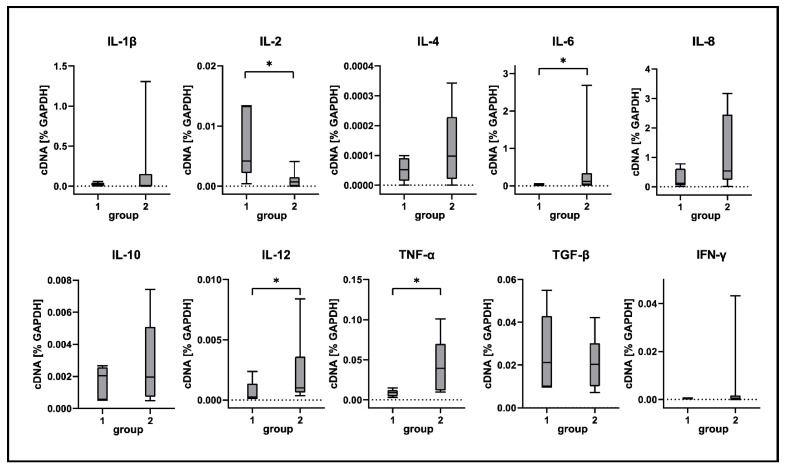
Cytokine expression analyses of canine distemper virus (CDV)-infected lung tissue. IL = interleukin; TNF-α = tumor necrosis factor alpha; TGF-β = transforming growth factor beta; IFN-γ = interferon gamma. Groups: 1 = non-infected control lungs; 2 = acute CDV infection; box and whisker plots display median and quartiles with maximum and minimum values. Significant differences (*p* ≤ 0.05, Kruskal–Wallis *H* test) are labeled by asterisks.

**Figure 9:**
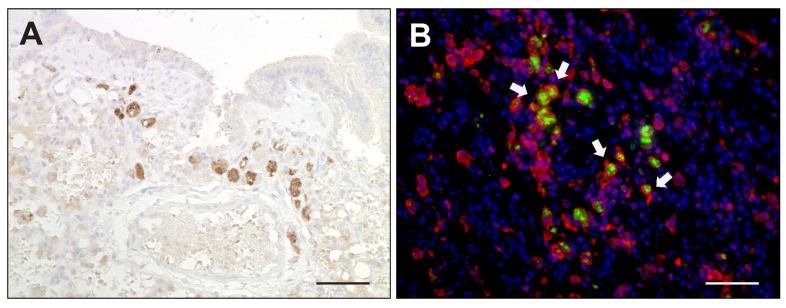
TNF-α expression in canine distemper virus-infected lungs. (**A**) Detection of TNF-α in mononuclear cells by immunohistochemistry. (**B**) Double immunofluorescence labeling against TNF-α (green) and Iba-1 (red) confirmed histiocytic cells (arrows) as a source of TNF-α. Nuclei were counterstained with bisbenzimide (blue). Scale bars: 50 µm.

Selected differences detected by RNA-Seq were confirmed by RT-qPCR and immunostaining. In agreement with RNA-Seq data, RT-qPCR analysis revealed a significant increase of TNF-α, IL-6 and IL-12 transcription in CDV-infected lungs compared to tissue from control animals (Figure 8). TNF-α expression was detected around bronchioles and within alveolar regions by immunohistochemistry. Immunofluorescence revealed Iba-1^+^ macrophages as a source of TNF-α production within lungs of CDV-infected dogs (Figure 9). Spearman correlation analyses revealed a positive correlation between CDV RNA loads and IL-6 (correlation coefficient: 0.560) and TNF-α transcription (correlation coefficient: 0.633). Lung IL-2 mRNA transcription was significantly downregulated in CDV-infected dogs, while no significant changes between groups were found for IL1-β, IL-4, IL-8, IL-10, TGF-β and IFN-γ mRNA expression (Figure 8).

### 2.6. Apoptosis Induction in Canine Distemper Virus-Infected Lungs

**Figure 10:**
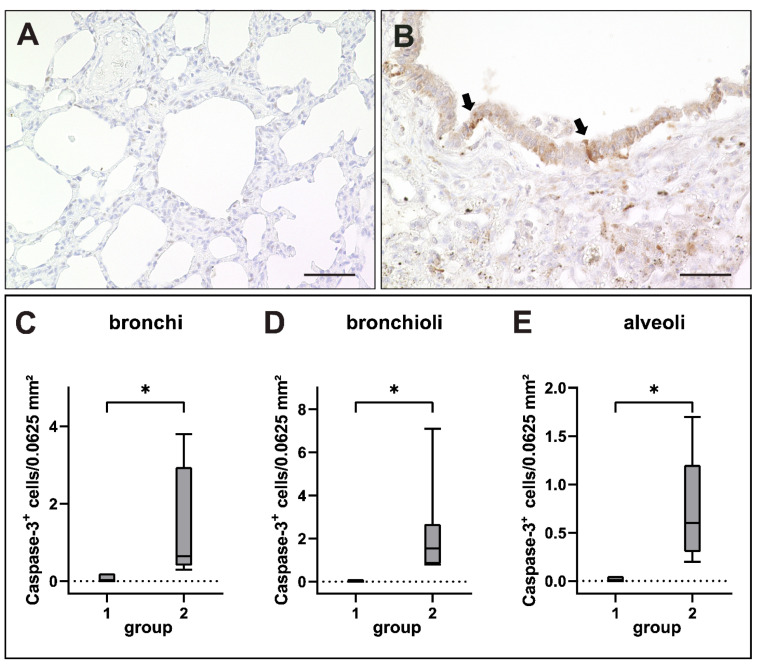
Apoptosis in canine distemper virus (CDV)-infected lungs. (**A**) Non-infected control lung. (**B**) Lung of a dog with CDV infection with CC-3^+^ apoptotic cells within the bronchiolar epithelium (arrows). (**C**–**E**) Increased numbers of apoptotic cells in bronchi (**C**), bronchioli (**D**) and alveoli (**E**) of infected dogs. Groups: 1 = non-infected control lung; 2 = acute CDV infection; box and whisker plots display median and quartiles with maximum and minimum values. Significant differences (*p* ≤ 0.05, Kruskal–Wallis *H* test) are labeled by asterisks. Scale bars: (**A**,**B**): 50 µm.

Quantification of immunohistochemical staining against cleaved caspase-3 (CC-3) confirmed an increase of apoptotic events as indicated by RNA-Seq data. Scattered CC-3^+^ apoptotic cells were found within airway and alveolar epithelia as well as within airway lumina of CDV-infected dogs (Figure 10).

### 2.7. Interferon-Related Genes

**Figure 11:**
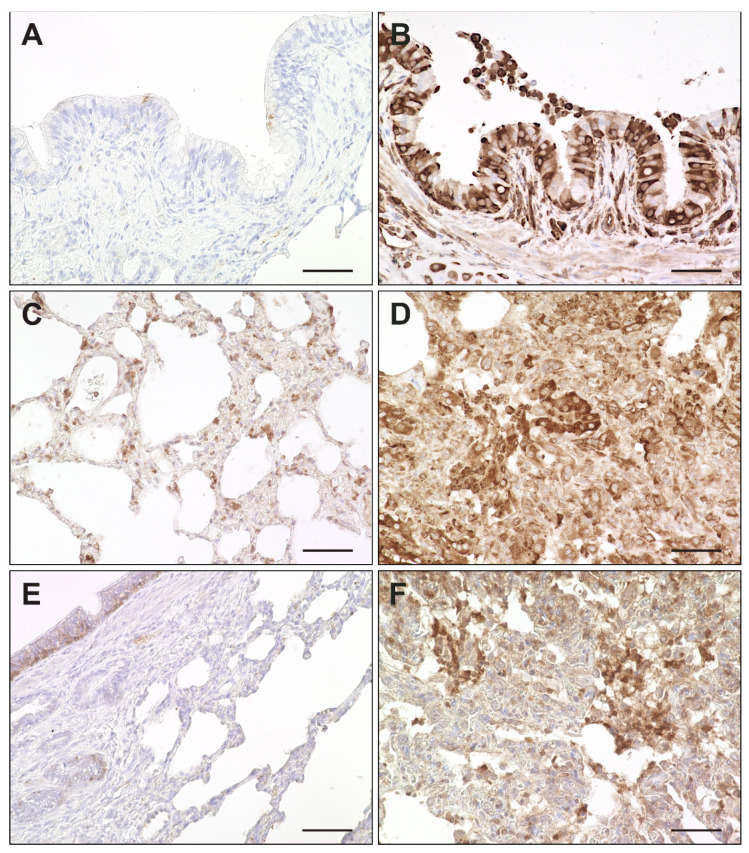
Expression of interferon-related genes in canine distemper virus (CDV)-infected lungs. (**A**,**B**) Mx protein in a non-infected (**A**) and acutely CDV-infected lung (**B**). (**C**,**D**) Protein kinase R (PKR) antigen detection in a non-infected (**C**) and acutely CDV-infected lung (**D**). (**E**,**F**) Interferon-simulated gene 15 (ISG15) antigen detection in basal bronchial epithelium of a non-infected lung (**E**) and in an acutely CDV-infected lung (**F**). Scale bars: 50 µm.

**Figure 12:**
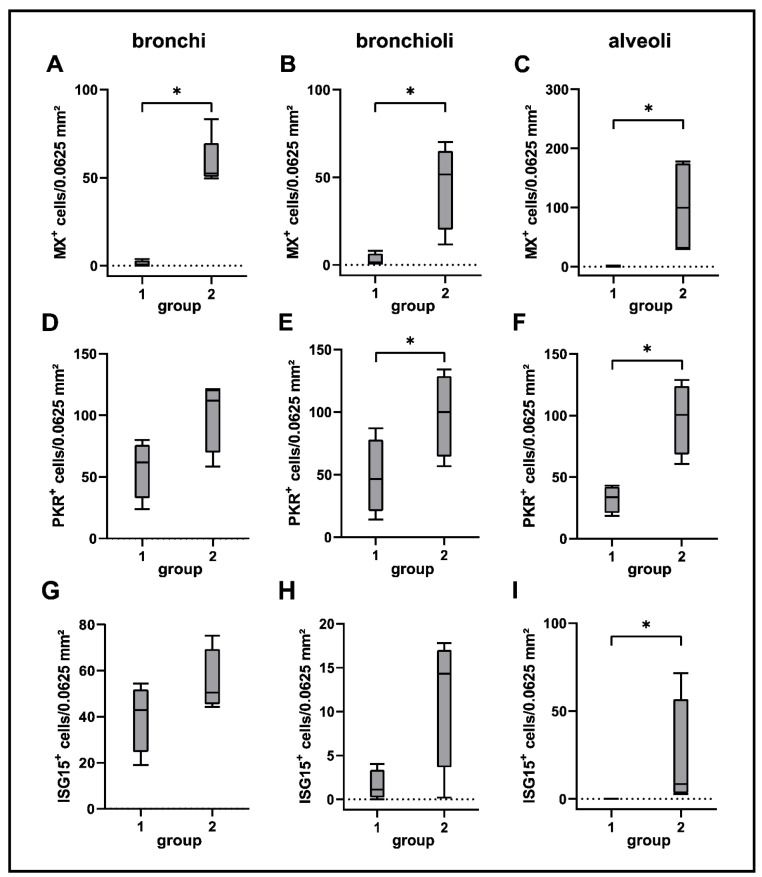
Quantification of Mx (**A**–**C**), PKR (**D**–**F**) and ISG15 (**G**–**I**) in lung tissue of canine distemper virus (CDV)-infected dogs. Groups: 1 = non-infected control lungs; 2 = acute CDV infection; box and whisker plots display median and quartiles with maximum and minimum values. Significant differences (*p* ≤ 0.05, Kruskal–Wallis *H* test) are labelled by asterisks.

Activation of interferon-related responses obtained by RNA-Seq analysis was confirmed by immunohistochemistry for interferon-induced proteins Mx, PKR and ISG15. ISG15 was constitutively expressed in basal cells of the bronchial epithelium and to a lesser extent in bronchial glands of non-infected dogs. Upon CDV infection, airway epithelial cells, pneumocytes and alveolar macrophages showed an increased expression of Mx, PKR and ISG15. Most prevalent changes were detected in alveolar regions of infected dogs. (Figure 11 and Figure 12).

## 3. Discussion

CDV infection causes pulmonary infection and pathology in susceptible hosts. The present study showed a transient damage of respiratory epithelia and pulmonary immune responses dominated by innate immune cells in affected dogs. Transcriptome analyses of lung tissue revealed the expression of several genes and pathways involved in antiviral immunity and control of virus infection, respectively.

In agreement with previous reports, pathologic findings in CDV-infected lungs are characterized by interstitial pneumonia with necrotizing bronchiolitis, syncytia formation and viral inclusion bodies [34,61,62]. Transcriptome analysis revealed a downregulation of genes involved in ciliary and epithelial function (cluster 4 and 5), which is in accordance with the observed epithelial pathology in investigated dogs. Disturbed mucociliary transport, either generated by the pathogen itself or excessive immune responses, inhibits self-clearance of airways and protection against invading pathogens, and has been demonstrated in mice and murine air–liquid interface cultures infected with paramyxoviruses (Sendai virus) [63,64]. Not only the pathogen itself, but also host responses, e.g. the production of reactive oxygen species (ROS) by inflammatory cells, have been shown to exhibit a negative impact on ciliary function [65]. Decreased inflammatory changes and pulmonary damage were found in the subacute-chronic phase of canine distemper, indicative of disease remission and viral elimination during the advanced infection phase. Accordingly, the number of CDV-infected cells transiently increased during the acute and subacute phase and only residual infection was present in the lung of dogs with subacute-chronic infection. Similarly, reduced viral loads have been shown in the spleen and brain of dogs during subacute-chronic CDV infection in previous studies [56,66,67]. Noteworthy, the reduction of inflammatory and pathologic changes in the lung contrasts with findings in the CNS of CDV-infected dogs, where inflammation progresses in the subacute-chronic phase despite of decreased viral burdens, indicating an immune-mediated neurologic disorder in the advanced infection phase [51,67,68]. Hence, the correlation between viral loads and severity of interstitial pneumonia found in the present study clearly indicates a primarily virus-mediated lung pathology in canine distemper and effective pulmonary antiviral immunity.

The innate immune system is the first line of defense against pathogens and plays a major role for antigen presentation and induction of virus-specific adaptive immunity [49,69]. Results of immune cell phenotyping indicate a local activation of innate immune responses in bronchial, bronchiolar and alveolar regions of CDV-infected dogs during the acute and subacute infection phase, with MHC-II expression by immune cells and pulmonary epithelium as well as a dominance of Iba-1^+^/CD204^+^ histiocytes. This process is assumed to be a consequence of pro-inflammatory cytokine expression, detected by transcriptome analysis and RT-qPCR, which enhances pulmonary recruitment of mononuclear cells and antigen presenting capacity [42]. In the lung, type II pneumocytes and airway epithelial cells, as part of the primary barrier, were reported to express MHC-II, albeit not as efficiently as professional antigen-presenting cells [70,71,72,73,74]. MHC-II upregulation by resident and infiltrating cells is a frequent finding in morbillivirus infection. For instance, it has been observed in CDV- and measles virus-infected brains as well as in lungs of CeMV-infected dolphins [41,51,53,60,75,76]. In chronic CNS lesions of CDV-infected dogs, MHC-II expression remained upregulated despite strongly reduced CDV loads, suggesting a trigger function of non-viral antigens for persistent neuroinflammatory responses [51].

Robust adaptive immune responses are crucial for viral elimination and disease recovery as well as protection from reinfection by induction of an immunological memory. Morbilliviruses target lymphoid cells, which causes depletion of lymphoid organs and long-lasting immunosuppression [25,77,78]. In the present study, a minor but significant infiltration of CD3^+^ T cells was observed in alveolar interstitial regions upon CDV infection, likely in response to pulmonary innate immune responses. By contrast, B cell numbers initially decreased within alveolar regions upon infection, which can be explained by the marked lymphotropism of CDV and induction of lymphoid apoptosis [79,80]. B cell depletion has been described also in the bronchus-associated lymphoid tissue (BALT) of measles patients and in measles virus-infected macaques, leading to B cell exhaustion and impaired humoral responses [59,81]. In the subacute-chronic phase of CDV infection, B cell repopulation was observed in lung samples, as previously described in CDV-infected lymphoid tissues [26,82]. Similarly, measles virus-infected macaques show transient leukopenia and subsequent restoration of peripheral lymphocyte populations [81]. Repopulation of lymphocytes indicates an intact proliferative capacity of lymphoid cells during the convalescent phase. However, these immune cells are supposed to be virus-specific and bystander lymphocytes, masking a depletion of pre-existing memory lymphocytes, which accounts for prolonged immune suppression and enhanced susceptibility for secondary infections after virus clearance [81]. Moreover, ex vivo experiments revealed that peripheral blood lymphocytes of measles patients fail to respond to expansion stimuli and show altered cytokine profiles during and after acute virus infection, indicating virus-induced T cell silencing despite normal lymphocyte counts (aka measles paradox) [83,84].

In order to obtain a more detailed view on pulmonary immune mechanisms in canine distemper, transcriptome analysis of lung tissue was performed. Data revealed a preferential regulation of genes related to antiviral defense mechanisms, including altered cytokine expression, cell death processes and interferon type I-related pathways. Cytokines orchestrate innate and adaptive immune responses in infectious disorders, while imbalanced cytokine expression leads to organ dysfunction and immunopathology [42,85,86]. Molecular analysis demonstrated an enhanced pro-inflammatory cytokine response, including TNF-α, IL-6 and IL-12 transcription, in lungs infected with CDV. TNF-α is produced mainly by macrophages and activated T cells in response to various stimuli, which is pivotal for protective antiviral immunity, but on the other hand potentially fosters immune mediated tissue damage [85,87,88]. Its beneficial effects in viral infection include the induction of antiviral immune responses by recruiting inflammatory cells and inhibiting viral replication, either directly or by inducing cell death of infected cells [46,89,90,91]. In the lung, TNF-α regulates epithelial sodium channels in type II pneumocytes and alveolar edema development [92,93,94,95,96]. Another destructive effect of TNF-α is disruption of the alveolar epithelial barrier by death signaling [97]. TNF-α also contributes to leakage of the endothelial barrier, via induction of reactive oxygen species (ROS) production [98,99,100,101] or rearrangement of microtubules [102,103,104]. The pro-oxidative effect of TNF-α is supported by its direct inhibition of the antioxidant glutathione [105]. In the present study, double labeling showed TNF-α expression by Iba-1^+^ cells in CDV-infected lungs, indicating a pro-inflammatory M1 phenotype of pulmonary macrophages in canine distemper. Increased TNF-α levels have been reported also within early brain lesions of CDV-infected dogs and are thought to enhance immune mediated damage demyelination in the CNS [41,53,106]. In the spleen, TNF-α expression might induce lymphocyte death during acute CDV infection [56,107]. Similar to CDV-infected tissues, TNF-α levels are increased also in measles virus-infected human glial cells and have been demonstrated in the spleen, lung and brain of measles virus-infected children [108,109].

IL-6 and IL-12 are secreted by macrophages and dendritic cells in response to virus infection. In concert with TNF-α, they initiate Th1 responses of CD4^+^ T cells, which are supposed to trigger CNS immunopathology during CDV infection [41,106,110,111]. Interestingly, IL-2 was shown to be downregulated in CDV-infected lungs. IL-2 is vital for memory T cell development, and its absence contributes to the loss of immune memory and a prolonged immunosuppressive state [112,113]. Moreover, IL-2 plays a major role in Foxp3^+^ regulatory T cell function, maintaining self-tolerance and preventing immunopathology [56,114,115]. Reduced IL-2 transcription and disturbed T cell function is observed also in spleens during acute CDV infection of dogs [56]. Similarly, deficient IL-2 production in peripheral blood mononuclear cells was found during measles virus infection [84]. Of note, expression of programmed death ligand-1 (PDL-1) was found in CDV-infected lungs by transcriptome analysis (cluster 1) in the present study, which acts as negative regulator of T cell effector responses and IL-2 transcription [116,117]. Interestingly, blockage of the programmed cell death-1/PDL-1 checkpoint pathway by measles virus enhances effector memory T cell response in vitro and in mouse models, representing a potential target for immunotherapy [118,119,120]. Noteworthy, immunomodulatory cytokines such as IL-4, IL-10 and TGF-β showed no significant changes in CDV-infected lung tissue in the present study. These cytokines suppress M1 macrophage functions and are involved in disease remission and tissue repair [121,122,123]. Similarly, previous reports have shown that IL-4, IL-10 and TGF-β expression is limited during early CDV infection in the CNS [41,53]. In addition, no increased expression of these cytokines was detected in morbillivirus-infected lungs of cetaceans [60]. These observations support the hypothesis of an imbalanced cytokine response towards a pro-inflammatory environment in canine lungs upon CDV infection.

During viral infections, apoptosis poses an antiviral defense response that results in elimination of infected cells, thereby inhibiting viral replication and enhancing adaptive immunity [124,125]. However, lymphoid cell death also contributes to leukopenia and immunosuppression during morbillivirus infections [79,80,126]. The present study revealed increased apoptotic events, most prominent within airway epithelia. Enhanced caspase 3 activation and apoptotic cell death were detected also in the CNS and lung of dolphins following CeMV infection [60]. Apoptosis induction has been reported in vitro directly by morbillivirus infection and indirectly in bystander cells by the release of toxic factors [126,127,128,129,130].

Transcriptome analysis also revealed an activation of interferon I pathways with an upregulation of interferon-related genes (IRGs). Distributions of Mx, PKR and ISG15 expressing cells in lung tissue were determined by immunohistochemistry. Type I interferons are key contributors to an effective innate antiviral response, which are also involved in modulation of cell differentiation and growth, the promotion of apoptosis, positive regulation of adaptive immune responses [131,132]. By binding to their receptors on host cells, they induce a signaling cascade, resulting in the production of IFN-stimulated proteins, such as Mx proteins, ISG15, protein kinase R and RNAse L [133]. Mx proteins appear to induce antiviral activity by interference with viral replication after sensing of viral nucleocapsid-like structures, as has been demonstrated in several RNA virus infections, including bunyavirus, influenza virus and measles virus infection [134,135,136,137]. In respiratory epithelial cells, it triggers early inflammatory responses following influenza virus infection [138]. Elevated Mx protein expression can also be found in the CNS of CDV-infected dogs [55,139]. Interestingly, toothed whales are devoid of Mx proteins, suggesting a constrained antiviral response to morbillivirus infection [140]. ISG15 is a ubiquitin-like protein, either inhibiting or activating diverse signaling cascades, such as NF-κB and retinoic acid-inducible gene I (RIG-I) pathways by protein-binding (“ISGylation”) [141,142]. Its effect varies among different species [143]. Antiviral activity by interference with viral replication has been suggested for pulmonary canine influenza virus infection and CDV brain infection [55,144]. Constitutive expression of ISG15 has been found in basal cells of the bronchial epithelium in the present study. Similarly, ISG15 is expressed in neurons and endothelial cells of non-infected canine cerebella, suggesting a function in normal protein turnover [55]. Protein kinase R inhibits viral and cellular mRNA translation via phosphorylation of eukaryotic translation initiation factor 2 alpha (eIF2α) [145,146]. By in vitro studies, it has been proposed to promote apoptosis [147]. The ISG RNAse L, which was upregulated in CDV-infected lungs, is an enzyme involved in degradation of cellular and viral RNA [148,149]. Additionally, RNAse L prevents viral entry into the host cell via interference with the cytoskeleton and propagates type I IFN response via a positive feedback mechanism by stimulation of the type I interferon inducing RIG-I pathway by its cleaved products [150,151]. Several viruses have been reported to antagonize RNAse L as an immune evasion strategy, including respiratory syncytial virus, influenza A virus and Theiler’s murine encephalomyelitis virus [149,152]. Taken together, these findings are comparable with analyses of IFN-related genes during natural CDV infection in the canine CNS [55,139]. In CDV-infected canine air–liquid interface cultures, an induced disturbance of interferon signaling by blockage of the interferon-induced JAK/STAT pathway enhanced cytopathic effects and facilitated viral spread, underlining the important role of type I interferons in the defense against respiratory CDV infection [153]. These results highlight the potential of interferon-based antiviral therapy in CDV infection, as shown by in vitro studies [154]. However, morbilliviruses also developed strategies to evade the IFN response. For instance, viral V protein of CDV and MV interfere with mda-5 and STATs, important regulator molecules of the IFN response [155,156,157,158]. The variable ability of morbilliviruses to inhibit the IFN response might be explained by the influence of different factors, including viral strain, infected species, infected tissue type, interferences with other signaling pathways and differences between in vivo and in vitro systems [132,159,160,161].

In conclusion, the present study delineates spatiotemporal phenotypic changes and lesion development in the canine lung, as well as molecular aspects of the antiviral response to CDV infection. The findings indicate the development of a pro-inflammatory environment driven by innate immune responses and impaired lymphocyte functions. The study represents the first report of CDV-related immune responses in lungs of its natural host, and lines up with previous studies in other target organs, which have shown a pro-inflammatory and potentially harmful immune response during CDV infection. The exploratory study contributes to an augmented understanding of morbillivirus pathogenesis and expands the list of immunologic parameters potentially contributing to viral elimination and virus-induced pulmonary immunopathology in canine distemper, providing a broad basis for further mechanistic research.

## 4. Materials and Methods

### 4.1. Ethical Statement

The present study was conducted in accordance with the German Animal Welfare Act. The authors confirm that no animals were experimentally infected or sacrificed for the purpose of this retrospective pathological study. All dogs used in the present study were dead at the time of submission to routine necropsy service. Some of the control tissues were collected from dogs deriving from an animal experiment, which was approved and authorized by the local authorities (Niedersächsisches Landesamt für Verbraucherschutz und Lebensmittelsicherheit (LAVES), Oldenburg, Germany, permission number 08A580). All dog owners provided written consent for the collection of the dogs’ tissue and its usage for research purposes.

### 4.2. Animals, Tissue Samples and Processing

Lung tissues from 37 dogs with natural CDV-infection (groups 2–4) and 7 non-infected control dogs (group 1) were examined by histology, immunohistochemistry and immunofluorescence. Snap frozen lung tissues from nine CDV-infected dogs and six non-infected control dogs were used for molecular analyses (bulk RNA sequencing and RT-qPCR). Clinical signs of CDV-infected dogs included seizures, ataxia, diarrhea, vomitus, dyspnea, coughing, fever and occasionally nasodigital hyperkeratosis. They either died spontaneously or were euthanized due to poor prognosis. CDV infection was confirmed post mortem via immunohistochemistry. Age, sex, breed and mode of death of dogs are listed in Supplementary Tables S3 and S4. During necropsy, lung tissue was collected and either immersion fixed in 10% neutrally buffered formalin or mounted in O.C.T.™ embedding compound (Tissue Tek^®^; Sakura Finetek Europe, Alphen aan den Rijn, The Netherlands) and stored at −80 °C until use for molecular analyses. To generate formalin-fixed and paraffin embedded slides, formalin-fixed tissue was dehydrated by ascending series of alcohols and subsequently embedded in paraffin (Thermo Fisher Scientific, Langenselbold, Germany). Then, 2–4 µm thick serial sections of lung tissue and cerebellum were cut with a rotary microtome (Leica Biosystems, Wetzlar, Germany), mounted on Superfrost^®^Plus slides (Gerhard Menzel, Braunschweig, Germany) and either stained with hematoxylin and eosin (HE) for histological evaluation or subjected to immunohistochemistry or immunofluorescence.

### 4.3. Classification of Disease Phases

Disease phases were determined based on the type of white matter lesions in the cerebellum. As shown before by experimental infections, CDV-induced leukoencephalitis in dogs develops in a sequential order. Acute lesions, characterized by white matter vacuolization and glial infection in the cerebellum, can be observed 16–24 days post infection. Subacute lesions with demyelination but without perivascular lymphohistiocytic cuffs occur 24–32 days after infection. Subacute to chronic lesions with demyelination together with perivascular lymphohistiocytic cuffs and reduced numbers of CDV^+^ cells can be found after a minimum of 29–63 days post infection in the brain of infected dogs [37,162,163,164,165,166,167]. Accordingly, dogs were classified into four groups: group 1 consisted of non-infected dogs without CNS disease (control); group 2 included dogs with cerebellar lesions with focal vacuolization and gliosis (acute phase); group 3 comprised dogs with demyelinating encephalitis without perivascular mononuclear infiltrates (subacute phase); group 4 included dogs with demyelinating encephalitis and perivascular mononuclear cuffing (subacute-chronic phase) [56,66].

### 4.4. Histological Scoring of Lung Lesions

HE-stained lung sections were evaluated independently by four scientists (E.C., P.P., J.K. and A.B.) for the intensity of inflammatory changes (mononuclear cell infiltration, type II pneumocyte hypertrophy and hyperplasia, bronchial/bronchiolar necrosis, fibrin extravasation and edema) and the presence of syncytia and viral inclusion bodies by light microscopy (Carl Zeiss, Jena, Germany). A semi-quantitative score was generated from the severity and extent of interstitial pneumonia as follows: 0 = no changes, 1 = severity: mild changes/extent: >25% of section affected, 2: severity: moderate changes/extent: 25–50% of section affected and 3: severity: marked changes/extent: >50% of section affected. Scores of both parameters were added.

### 4.5. Immunohistochemistry

CDV antigen detection and phenotyping of cellular immune responses was performed using the avidin–biotin complex method as described previously with slight modifications [51]. For ISG15, the EnVision visualization system was used. Respective primary antibodies are detailed in Table 2. In brief, formalin-fixed and paraffin-embedded (FFPE) slides were deparaffinized by ROTICLEAR^®^ (Carl Roth, Karlsruhe, Germany) and rehydrated through a series of graded alcohols for 2–3 min each. To suppress endogenous peroxidase activity, they were incubated with H_2_O_2_ (0.5%) in 85% ethanol for 30 min, followed by antigen retrieval in citrate buffer (pH 6.0) for 20 minutes in a microwave (800 W). Unspecific reactions with the secondary antibody were blocked by incubation with goat normal serum for 20 min at room temperature (except ISG15). The primary antibodies (CDV-NP, MHC-II, Iba-1, CD204, CD3, CD20, TNF-α, cleaved caspase-3 (CC-3), Mx, PKR and ISG15), diluted in phosphate-buffered saline (PBS) with 1% bovine serum albumin (BSA, Carl Roth GmbH), were incubated overnight (18 h) at 4 °C. Negative controls were treated with ascites fluid from non-immunized BALB/c mice (CDV-NP, MHC-II, CD204, TNF-α and Mx) or serum from non-immunized rabbits (Iba-1, CD3, CD20, CC-3, PKR and ISG15) instead of the primary antibody. As positive controls, immunohistochemically confirmed CDV-positive canine lung and cerebellar tissue (CDV-NP, Mx, PKR and ISG15) and canine lymph node tissue (MHC-II, Iba-1, CD204, CD3, CD20, TNF-α and CC-3) were used. Secondary labeling was performed using polyclonal biotinylated antibodies diluted 1:200 in PBS (goat anti-mouse, Vector Laboratories, Burlingame, CA, USA, BA-9200; goat anti-rabbit, Vector Laboratories, BA-1000) for 45 min at room temperature, followed by a treatment with the avidin–biotin–peroxidase complex (Vectastain Elite ABC Kit, Vector Laboratories) for 20 min at room temperature. For ISG15, the EnVision+ anti-rabbit HRP-labelled polymer antibody (Dako, Glostrup, Denmark; K4003) was incubated for 30 min at room temperature instead of using the biotinylated antibodies and the avidin–biotin–peroxidase complex. Subsequently, the reaction was visualized by incubation in 0.05% 3.3′-diaminobenzidine tetrahydrochloride (DAB, Carl Roth) in PBS and H_2_O_2_ (0.03%) for 5 min at room temperature. Nuclei were counterstained with Mayer’s hemalum solution (Carl Roth) for 30 s with subsequent dehydration in 70% ethanol, 96% ethanol, isopropanol and n-Butyl acetate.

Immunoreactivity in lung sections was evaluated quantitatively by counting absolute numbers of positive cells using a morphometric grid (number of positive cells/0.0625 mm^2^) in 10 randomly chosen high power fields within affected and control lungs. Subsequently, the mean value of immunopositive cells per 0.0625 mm^2^ was calculated.

### 4.6. Immunofluorescence Double Labeling

To determine the cell tropism of CDV in infected lungs, immunofluorescence labeling of CDV nucleoprotein, either combined with Iba-1 (histiocytic cell antigen) of pan-cytokeratin (CK, epithelial cell antigen) was performed on lung tissue of 12 CDV-infected dogs. Additionally, immunofluorescence was applied to demonstrate TNF-α production in Iba-1^+^ macrophages in lung sections (TNF-α/Iba-1 double labeling). Used primary antibodies are listed in Table 2. Following deparaffinization by ROTICLEAR^®^ (Carl Roth), rehydration through graded alcohols and rinsing in PBS with a stirring tool (3 × 5 min), the slides were either pretreated with citrate buffer (pH 6.0, CDV/Iba-1 and CDV/CK) for 20 min or Tris-EDTA buffer (pH 9.0, TNF-α/Iba-1) for 30 min in a microwave (800 W). Unspecific bindings of the secondary antibody were blocked by 20% goat normal serum in PBS with 1% BSA and 0.1% Triton-X100 (Sigma-Aldrich, St. Louis, MO, USA) for 30 min. Both primary antibodies were diluted simultaneously in PBS with 1% BSA and 0.1% Triton X-100 and were incubated overnight (18 h) at 4 °C. Negative controls were treated with ascites fluid from non-immunized BALB/c mice and rabbit normal serum instead of the primary antibodies. After washing thrice in PBS, the secondary polyclonal antibodies (Alexa Fluor^®^ 488-conjugated goat anti-mouse (Jackson ImmunoResearch Europe, Ely, UK, 115-545-003) and Cy3-conjugated goat anti-rabbit (Jackson ImmunoResearch Europe, 111-165-144)) were diluted at 1:200 in PBS with 1% BSA and 0.1% Triton X-100 and subsequently incubated for 45 minutes at room temperature in the dark, followed by rinsing in PBS for 3 × 5 min. The slides were washed twice with distilled water preceding an autofluorescence-reduction treatment (Vector TrueVIEW Autofluorescence Quenching Kit, Vector Laboratories, Burlingame, CA, USA) for 5 min, followed by rinsing thrice with PBS and twice with distilled water. Nuclei were counterstained with bisbenzimide Hoechst 33,258 (1:100 in sterile bidistilled water; Sigma-Aldrich Chemie, Taufkirchen, Germany) for 8 min, and slides were mounted with fluorescence mounting medium (Dako, Glostrup, Denmark), followed by curing and storage in the dark at 4 °C until inspection.

Evaluation of colocalization was performed with a fluorescence microscope (Keyence BZ-9000E with BZ-II-analyzer software BZ-H2AE, Keyence, Mechelen, Belgium) equipped with hard-coated band pass filters for bisbenzimide (emission 447/60 nm, excitation 377/55 nm), Cy3 (emission 624/40 nm, excitation 562/40 nm) and AlexaFluor^®^488 (emission 520/35 nm, excitation 472.5/30 nm), respectively.

### 4.7. Molecular Investigations

In order to investigate immune responses and CDV RNA loads on a molecular level, RNA sequencing analysis and quantitative reverse transcription polymerase chain reaction (RT-qPCR) were performed.

#### 4.7.1. RNA Isolation

OCT-embedded frozen lung tissue from CDV-infected dogs and non-infected control dogs as well as frozen lymph node tissue from healthy dogs (used for controls) was cut with a cryostat microtome at 50 µm (Leica CM1950, Leica Biosystems Nussloch GmbH, Nussloch, Germany). Isolation and purification of total RNA was achieved using the RNeasy^®^ Mini Kit (Qiagen, Hilden, Germany), according to the manufacturer’s instructions, including an on-column DNAse treatment (Qiagen). The obtained RNA amount was calculated by measuring the optical density at 260 nm with a spectrophotometer (Multiskan^TM^ GO microplate spectrophotometer, µDrop^TM^ plate, SkanIt^TM^ software version 5.0.0.42, Thermo Fisher Scientific, Braunschweig, Germany).

#### 4.7.2. RNA Sequencing Analysis

For analysis of transcriptional changes during CDV-infection in the lung, the isolated RNA of four control animals and five acutely infected dogs was selected. Quality and integrity of total RNA was controlled on Agilent Technologies 2100 Bioanalyzer (Agilent Technologies, Waldbronn, Germany). The RNA sequencing library was generated from 50ng rRNA depleted total RNA (QIAseq FastSelect rRNA HMR, Qiagen) using NEB Ultra II Directional-RNA Seq Library-Kit (New England Biolabs, Ipswich, MA, USA) according to the manufacture’s protocol. The libraries were sequenced on Illumina NovaSeq 6000 using NovaSeq 6000 S1 Reagent Kit (100 cycles, paired end run) with an average of 5 × 10^7^ reads per RNA sample. Each FASTQ file obtained a quality report generated by FASTQC tool. Before alignment to the reference genome, each sequence in the raw FASTQ files was trimmed on base call quality and sequencing adapter contamination using Trim Galore! wrapper tool. Reads shorter than 20 bp were removed from the FASTQ file and trimmed reads were aligned to the reference genome (Canis_lupus_familiaris.ROS_Cfam_1.0, https://ensemblgenomes.org, accessed on 22 March 2022) using open source short read aligner STAR (https://code.google.com/p/rna-star, accessed on 22 March 2022) with settings according to log file. Feature counts were determined using the R package Rsubread (version 2.10.5, available at https://doi.org/doi:10.18129/B9.bioc.Rsubread, accessed on 22 March 2022) [168]. Data were cleansed by considering only genes showing counts greater 5 at least two times across all samples for further analysis. Gene annotation was conducted by the R package bioMaRt (version 2.52.0, available at https://doi.org/doi:10.18129/B9.bioc.biomaRt, accessed on 22 March 2022) [169,170]. Expression data was log2 transformed and TMM normalized followed by calculation of differential gene expression by the R package edgeR (version 3.38.4, available at https://doi.org/doi:10.18129/B9.bioc.edgeR, accessed on 22 March 2022) [171,172,173]. Differential expressed genes (DEGs) were defined as genes with *p* values < 0.05. Functional analysis was performed by the R package clusterProfiler (version 4.4.4, available at https://doi.org/doi:10.18129/B9.bioc.clusterProfiler, accessed on 22 March 2022) [174,175].

#### 4.7.3. Reverse Transcription

Transcription of total RNA into complementary DNA (cDNA) was performed using the Omniscript^®^ Reverse Transcription Kit (Qiagen) with RNaseOUT^TM^ Recombinant Ribonuclease Inhibitor (Invitrogen, Carlsbad, CA, USA), and random primers (Promega Corporation, Madison, WI, USA), according to the manufacturer’s protocol.

#### 4.7.4. Primers and Plasmids

For generation of standard dilutions, primer sequences for qualitative PCR of glyceraldehyde-3-phosphate dehydrogenase (GAPDH), CDV, IL-2, IL-6, IL-8 and TNF-α were taken from the literature [41,176,177,178] (Table 3). For standard dilution of IL-4, IL-10, IL-12, transforming growth factor beta (TGF-β) and interferon gamma (IFN-γ), plasmid gene sequences < 300 bp length, including the sequence of the qPCR product were selected from the respective cDNA genome with pEX-A128 as a vector backbone (Table 4).

Primer sequences for quantitative RT-PCR detection of GAPDH, CDV, IL-2, IL-4, IL-6, TNF-α, TGF-β1 and IFN-γ were taken from the literature [176,177,179,180] (Table 5). The other primers were designed using the software tool Primer-BLAST using Primer3 and BLAST (https://www.ncbi.nlm.nih.gov/tools/primer-blast, accessed on 15 March 2021) [181]. All primers and plasmids were purchased from Eurofins Genomics (Ebersberg, Germany).

#### 4.7.5. Generation of Standard Dilutions

For production of standards via PCR, cDNA either derived from CDV-infected lung tissue (CDV, GAPDH, IL-1β and IL-8) or lymph nodes from non-infected dogs (IL-2, IL-6 and TNF-α) was amplified using the T-Gradient thermocycler (Biometra, Göttingen, Germany) as described previously. Annealing temperature was adjusted to 58 °C (TNF-α and IL-6) and 59 °C (GAPDH, CDV, IL-1-β, IL-2 and IL-8), respectively, at 45 s, followed by elongation at 72 °C for 40 s. Amplification was achieved using Taq DNA Polymerase (Invitrogen™, Thermo Fisher Scientific, Langenselbold, Germany) with 1.5 mmol/L MgCl_2_, 0.2 mmol/L dNTP mix (New England Biolabs) and 300 nmol/L of each primer. PCR products were subsequently visualized by agarose gel electrophoresis and extracted with NucleoSpin^®^ Gel and PCR Clean-up (Macherey-Nagel, Düren, Germany). Determination of DNA concentration was performed with a spectrophotometer at 260 nm (Multiskan^TM^ GO microplate spectrophotometer, µDrop^TM^ plate, SkanIt^TM^ software version 5.0.0.42, Fisher Scientific, Schwerte, Germany). Extracted PCR products and plasmids (dissolved in DNAse and RNAse-free water) were used for production of a standard dilution series from 10^8^ to 10^2^ copies/µL.

#### 4.7.6. Reverse Transcription Quantitative PCR (RT-qPCR) Analysis

Quantities of CDV cDNA loads and mRNA expression of cytokines in CDV-infected lung samples and uninfected controls were assessed together with the standard dilution series and no template controls via RT-qPCR using the AriaMx Real-Time PCR System (Agilent Technologies; Agilent Aria software version 1.71). The Brilliant III Ultra-Fast SYBR^®^ Green QPCR Master Mix (Agilent Technologies) was used following the manufacturer’s instructions with primers at a concentration of 200 nmol/L and carboxy-X-rhodamine (ROX) as a reference dye. Annealing temperatures were adjusted to 57 °C (CDV), 60 °C (IL-1-β, IL-2, IL-4, IL-6, IL-8, IL-10, IL-12, TNF-α, TGF-β and IFN-γ) or 64 °C (GAPDH). Copy numbers were calculated by comparing with the standard curves and were normalized against GAPDH as a housekeeping gene. Reaction specificity was assessed by melting curve analysis.

### 4.8. Statistical Analysis

Statistical analysis of non-normally distributed data obtained by immunohistochemistry and quantitative PCR was performed by employing the non-parametric Kruskal–Wallis *H* test for two independent samples using the IBM “Statistic Package for Social Sciences” SPSS program for Windows (version 26, SPSS^®^, IBM, Ehningen, Germany). To detect a possible correlation between CDV loads and interstitial pneumonia and the cytokine transcription, respectively, the Spearman rank correlation coefficient was calculated for all investigated variables. *p*-values ≤ 0.05 were designated as statistically significant when comparing differences between groups. Graphs were generated using GraphPad Prism^®^ for Windows (version 9.9.0, GraphPad Software, San Diego, CA, USA).

## Figures and Tables

**Table 1:** Selected significantly enriched gene ontology (GO) terms in clusters 1–5.

Gene Set	GO Terms (Biological Function)	Adjusted *p*-Value
DEGs in cluster 1 (n = 353)	defense response to virus	0.000
	negative regulation of viral process	0.001
	negative regulation of viral genome replication	0.000
	positive regulation of innate immune response	0.002
	leukocyte activation involved in immune response	0.028
	response to type I interferon	0.013
DEGs in cluster 2 (n = 515)	mononuclear cell proliferation	0.000
	regulation of leukocyte activation	0.000
	T-helper 1 type immune response	0.001
	positive regulation of cytokine production	0.000
	negative regulation of cytokine production	0.031
	cellular response to cytokine stimulus	0.000
	cytokine-mediated signaling pathway	0.000
	tumor necrosis factor superfamily cytokine production	0.010
	positive regulation of apoptotic signaling pathway	0.035
	regulation of apoptotic process	0.000
	receptor signaling pathway via STAT	0.000
DEGs in cluster 3 (n = 118)	no enriched GO terms	
DEGs in cluster 4 (n = 1373)	cilium assembly	0.000
	cilium movement	0.000
DEGs in cluster 5 (n = 893)	epithelial cell proliferation	0.040

DEGs = differentially expressed genes.

**Table 2:** Primary antibodies used for immunohistochemistry and immunofluorescence.

Epitope	Specificity	Source	Cat No	Species	Clone	Dilution
CDV-NP	CDV-infected cells	A. Zurbriggen, University of Bern, Bern, Switzerland	-	mouse	D110	1:1000 *
CDV-NP	CDV-infected cells	Santa Cruz Biotechnology, Dallas, TX, USA	sc-57660	mouse	DV2-12	1:100 **
MHC-II	antigen-presenting cells	Dako, Glostrup, Denmark	M0746	mouse	TAL.1B5	1:80 *
Iba-1	histiocytic cells	Invitrogen™, Thermo Fisher Scientific, Langenselbold, Germany	PA5-27436	rabbit	polyclonal	1:500 *; 1:200 **
CD204	histiocytic cells	Abnova Corporation, Taipei, Taiwan	MAB1710	mouse	SRA-E5	1:500 *
CD3	T lymphocytes	Dako, Glostrup, Denmark	A0452	rabbit	polyclonal	1:500 *
CD20	B lymphocytes	Lab Vision, Thermo Fisher Scientific, Langenselbold, Germany	RB9013P	rabbit	polyclonal	1:300 *
CK	epithelial cells	Dako, Glostrup, Denmark	Z0622	rabbit	polyclonal	1:50 **
TNF-α	TNF-α	Novus Biologicals, Centennial, CO, USA	NBP2-34303	mouse	TNF706	1:50 *; 1:20 **
CC-3	apoptosis	Cell Signaling Technology, Danvers, MA, USA	9664	rabbit	5A1E	1:200 *
Mx	antiviral immune response	O. Haller and G. Kochs, University Medical Center Freiburg, Freiburg, Germany	-	mouse	M143	1:800 *
PKR	antiviral immune response	Abcam, Cambridge, MA, USA	ab32036	rabbit	E120	1:600 *
ISG15	antiviral immune response	Boster Biological Technology, Pleasanton, CA, USA	PB9951	rabbit	polyclonal	1:750 *

Cat No = catalogue number; CDV-NP = canine distemper virus nucleoprotein; Iba-1 = ionized calcium-binding adapter molecule 1; MHC-II = major histocompatibility complex class II; CK = cytokeratin; TNF-α = tumor necrosis factor alpha; CC-3 = cleaved caspase 3 (Asp175); Mx = interferon-induced GTP-binding protein Mx; PKR = protein kinase R (phospho T446); ISG15 = interferon-stimulated gene 15; * dilution used for immunohistochemistry; ** dilution used for immunofluorescence. Pretreatment: heat-induced antigen retrieval in citrate buffer (pH 6.0) for 20 min or in Tris-EDTA-buffer (pH 9.0, for TNF-α/Iba-1 immunofluorescence double labeling) in a microwave (800 W).

**Table 3:** Primers used for qualitative RT-PCR for generation of standard dilutions.

Gene	Primer Direction	Primer Sequence (5′–3′)	Position	Amplicon Length (bp)	Acc No.	Reference
GAPDH	forward	AAG GTC GGA GTC AAC GGA TT	7–26	365	AB038240	Puff et al., 2008 [176]
	reverse	GCA GAA GGA GCA GAG ATG ATG	371–351			
CDV	forward	ACA GGA TTG CTG AGG ACC TAT	769–789	287	AF378705	Markus et al., 2002 [41]
	reverse	CAA GAT AAC CAT GTA CGG TGC	1055–1035			
IL-1-β	forward	TCC AAT GTG AAG TGC TGC TG	14–33	262	Z70047	Primer-BLAST [181]
	reverse	GCA TGG CTG CAT CAC TCA TA	275–256			
IL-2	forward	ACC TCA ACT CCT GCC ACA AT	14–33	289	D30710	Schwartz et al., 2011 [177]
	reverse	GCA CTT CCT CCA GGT TTT TG	302–283			
IL-6	forward	TCT CCA CAA GCG CCT TCT CC	68–87	318	U12234	Gröne et al., 1998 [178]
	reverse	TTC TTG TCA AGC AGG TCT CC	385–366			
IL-8	forward	ACT TCC AAG CTG GCT GTT GC	39–58	172	D28772	Gröne et al., 1998 [178]
	reverse	GGC CAC TGT CAA TCA CTC TC	210–191			
TNF-α	forward	CCA AGT GAC AAG CCA GTA GC	32–51	274	Z70046	Gröne et al., 1998 [178]
	reverse	TCT TGA TGG CAG AGA GTA GG	305–287			

bp = base pairs; Acc No = GenBank accession number; GAPDH = glyceraldehyde-3-phosphate dehydrogenase; CDV = canine distemper virus; IL = interleukin; TNF-α = tumor necrosis factor alpha.

**Table 4:** Plasmids used for generation of standard dilutions.

Gene	Plasmid Sequence (5’–3’)	Position	Gene Size (bp)	Acc No
IL-4	ACT GAT TCC AAC TCT GGT CTG CTT ACT AGC ACT CAC CAG CAC CTT TGT CCA CGG ACA TAA CTT CAA TAT TAC TAT TAA AGA GAT CAT CAA AAT GTT GAA CAT CCT CAC AGC GAG AAA CGA CTC GTG CAT GGA GCT GAC TGT CAA GGA CGT CTT CAC TGC TCC AAA GAA CAC AAG CGA TAA GGA AAT CTT CTG CAG AGC TGC TAC TGT ACT GCG GCA GAT CTA TAC ACA CAA CTG CTC CAA CAG ATA TCT CAG AGG ACT CTA CAG GAA CCT CAG CAG CAT GGC AAA CAA GA	81–370	290	AF239917
IL-10	ATG CCC CGG GCT GAG AAC CAC GAC CCA GAC ATC AAG AAC CAC GTG AAC TCC CTG GGA GAG AAG CTC AAG ACC CTC AGG CTG AGA CTG AGG CTG CGA CGC TGT CAC CGA TTT CTT CCC TGT GAG AAT AAG AGC AAG GCG GTG GAG CAG GTG AAG AGC GCA TTT AG	287–450	164	U33843
IL-12	ATG CAT CCT CAG CAG TTG GT C ATC TCC TGG TTT TCC CTC GTT TTG CTG GCG TCT TCC CTC ATG ACC ATA TGG GAA CTG GAG AAA GAT GTT TAT GTT GTA GAG TTG GAC TGG CAC CCT GAT GCC CCC GGA GAA ATG GTG GTC CTC ACC TGC CAT ACC CCT GAA GAA GAT GAC ATC ACT TGG ACC TCA GCG CAG AGC AGT GAA GTC CTA GGT TCT GGT AAA ACT CTG ACC ATC CAA GTC AAA GAA TTT GGA GAT GCT GGC CAG TAT ACC TGC CAT AAA GGA	1–279	279	U49100
TGF-β	GGA GCT GTA CCA GAA ATA TAG CAA TGA TTC CTG GCG CTA CCT CAG CAA CCG GCT GCT GGC GCC CAG CGA CAC GCC AGA ATG GCT GTC CTT TGA TGT CAC TGG AGT CGT GAG GCA GTG GCT GAG CCA TGG AGG GGA AGT CGA GGG CTT TCG CCT CAG TGC CCA CTG TTC CTG TGA CAG CAA AGA TAA CAC A	561–750	190	L34956
IFN-γ	CCA GAT GTA TCG GAC GGT GGG TCT CTT TTC GTA GAT ATT TTG AAG AAA TGG AGA GAG GAG AGT GAC AAA ACA ATC ATT CAG AGC CAA ATT GTC TCT TTC TAC TTG AAA CTG TTT GAC AAC TTT AAA GAT AAC CAG ATC ATT CAA AGG AGC ATG GAT ACC ATC AAG GAA GAC ATG CTT GGC AAG TTC TTA AAT AGC AGC ACC AGT AAG AGG GAG GAC TTC CTT AAG CTG ATT CAA ATT CCT GTG AAC GAT CTG CAG GTC CAG CGC AAG GCG ATA A	76–349	274	S41201

bp = base pairs; Acc No = GenBank accession number; IL = interleukin; TGF-β = transforming growth factor beta; IFN-γ = interferon gamma.

**Table 5:** Primers used for RT-qPCR.

Gene	Primer Direction	Primer Sequence (5′–3′)	Position	Amplicon Length (bp)	Acc No	Reference
GAPDH	forward	GTC ATC AAC GGG AAG TCC ATC TC	196–218	84	AB038240	von Smolinski et al., 2005 [179]
	reverse	AAC ATA CTC AGC ACC AGC ATC AC	279–257			
CDV	forward	GCT CTT GGG TTG CAT GAG TT	954–973	83	AF378705	Puff et al., 2008 [176]
	reverse	GCT GTT TCA CCC ATC TGT TG	1036–1017			
IL-1-β	forward	TGT CAG TCA TTG TAG CTT TG	123–142	113	Z70047	Primer-BLAST [181]
	reverse	GCA GAT GAT AGG TTC TTC TT	235–216			
IL-2	forward	CCA ACT CTC CAG GAT GCT CAC	196–216	81	D30710	Schwartz et al., 2011 [177]
	reverse	TCT GCT AGA CAT TGA AGG TGT GTA	276–252			
IL-4	forward	CTC CAA AGA ACA CAA GCG ATA AGG	239–262	84	AF239917	Schwartz et al., 2011 [177]
	reverse	TGT TGG AGC AGT TGT GTG TAT AGA	322–299			
IL-6	forward	TGA TGC CAC TTC AAA TAG TCT ACC A	156–180	89	U12234	Spitzbarth et al., 2011 [180]
	reverse	TCA GTG CAG AGA TTT TGC CGA GGA	244–221			
IL-8	forward	TTC GAT GCC AGT GTA TAA AA	130–149	74	D28772	Primer-BLAST [181]
	reverse	GTC AAT CAC TCT CAG TTC TT	203–184			
IL-10	forward	ACC ACG ACC CAG ACA TCA AGA A	303–324	120	U33843	Primer-BLAST [181]
	reverse	CCT TGC TCT TAT TCT CAC AGG GAA G	422–398			
IL-12	forward	CTC GTT TTG CTG GCG TCT TC	37–56	153	U49100	Primer-BLAST [181]
	reverse	CGC TGA GGT CCA AGT GAT GT	189–170			
TNF-α	forward	GGA GCT GAC AGA CAA CCA GCT GA	133–155	91	Z70046	Spitzbarth et al., 2011 [180]
	reverse	GGA AGG GCA CCC TTG GCC CT	223–204			
TGF-β	forward	TGG CGC TAC CTC AGC AAC CG	592–611	115	L34956	Spitzbarth et al., 2011 [180]
	reverse	AGC CCT CGA CTT CCC CTC CA	706–687			
IFN-γ	forward	AGC ATG GAT ACC ATC AAG GAA GA	223–245	104	S41201	Schwartz et al., 2011 [177]
	reverse	AGA TCG TTC ACA GGA ATT TGA ATC A	326–302			

bp = base pairs; Acc No = GenBank accession number; GAPDH = glyceraldehyde-3-phosphate dehydrogenase; CDV = canine distemper virus; IL = interleukin; TNF-α = tumor necrosis factor alpha; TGF-β = transforming growth factor beta; IFN-γ = interferon gamma.

## Data Availability

All relevant data are included in the manuscript, Supplementary Materials or can be obtained from the authors on reasonable request.

## Supplementary Materials

The following supporting information can be downloaded at: https://www.mdpi.com/article/10.3390/ijms231710019/s1.
