# Supplementary material for: Phenotypic and Transcriptional Changes of Pulmonary Immune Responses in Dogs Following Canine Distemper Virus Infection
Source: Int J Mol Sci. 2022 Sep 2;23(17):10019. doi: 10.3390/ijms231710019 (PMC9456005; doi:10.3390/ijms231710019)
Supplement: Supplementary file 1 [file ijms-23-10019-s001.zip › Supplementary_Tables_S3_S4_Chludzinski_et_al_25.08.22.pdf]

Supplementary material

Phenotypic and Transcriptional Changes of Pulmonary Immune Responses in Dogs Following Canine Distemper Virus Infection

Elisa Chludzinski[1,4], Johanna Klemens[1], Małgorzata Ciurkiewicz[1], Robert Geffers[2], Pauline Pöpperl[1,4], Melanie Stoff[1], Dai-Lun Shin[3], Georg Herrler[3,*], Andreas Beineke[1,4,*]

[1]Department of Pathology, University of Veterinary Medicine Hannover, 30559 Hannover, Germany
[2]Genome Analytics, Helmholtz Centre for Infection Research, 38124 Braunschweig, Germany
[3]Institute of Virology, University of Veterinary Medicine Hannover, 30559 Hannover, Germany
[4]Center for Systems Neuroscience (ZSN), 30559 Hannover, Germany

*Both authors have contributed equally last (G.H., A.B.)

Supplementary material:

This file includes: Supplementary Tables S3-S4

Supplementary Table S3. Anamnestic details of dogs used for evaluation of FFPE tissue

Dog No	CDV status	Group	Age [months]	Sex	Breed	Mode of death
**1**	non-infected	1	4.5	n.d.	Beagle	E
**2**	non-infected	1	4.5	n.d.	Beagle	E
**3**	non-infected	1	4.5	n.d.	Beagle	E
**4**	non-infected	1	136.0	f	Beagle	E
**5**	non-infected	1	n.d.	sf	Rhodesian Ridgeback	S
**6**	non-infected	1	109.0	sf	Bolonka Zwetna	E
**7**	non-infected	1	2	f	Greater Swiss Mountain Dog	E
**8**	CDV+	2	24.0	m	Mixed breed	E
**9**	CDV+	2	5.5	m	Old English Sheepdog	S
**10**	CDV+	2	3.0	w	Mixed breed	n.d.
**11**	CDV+	2	72.0	m	Mixed breed	E
**12**	CDV+	2	n.d.	f	n.d.	n.d.
**13**	CDV+	2	3.0	w	Mixed breed	n.d.
**14**	CDV+	2	6.0	w	Mixed breed	S
**15**	CDV+	2	n.d.	f	Mixed breed	n.d.
**16**	CDV+	2	3.0	m	Mixed breed	E
**17**	CDV+	2	3.5	f	Jack Russell Terrier	E
**18**	CDV+	2	3.0	f	English Bulldog	E

Dog No	CDV status	Group	Age [months]	Sex	Breed	Mode of death
**19**	CDV^{+}	2	2.0	f	Mixed breed	E
**20**	CDV^{+}	2	n.d.	f	n.d.	S
**21**	CDV^{+}	2	17.0	sf	Mixed breed	E
**22**	CDV^{+}	2	6.0	f	n.d.	E
**23**	CDV^{+}	3	n.d.	n.d.	German Shepherd	n.d.
**24**	CDV^{+}	3	4.0	m	German Shepherd	n.d.
**25**	CDV^{+}	3	n.d.	n.d.	n.d.	n.d.
**26**	CDV^{+}	3	3.0	f	Dachshund	S
**27**	CDV^{+}	3	6.0	sf	Mixed breed	n.d.
**28**	CDV^{+}	3	2.0	f	Labrador Retriever	E
**29**	CDV^{+}	3	2.0	m	Mixed breed	E
**30**	CDV^{+}	3	6.0	m	Mixed breed	S
**31**	CDV^{+}	3	3.0	m	Shih Tzu	S
**32**	CDV^{+}	3	5.0	f	Chihuahua	S
**33**	CDV^{+}	3	4.0	f	Mixed breed	E
**34**	CDV^{+}	4	4.0	f	Dachshund	n.d.
**35**	CDV^{+}	4	24.0	f	Mixed breed	n.d.
**36**	CDV^{+}	4	n.d.	m	German Shepherd	E
**37**	CDV^{+}	4	6.0	f	German Shepherd	n.d.
**38**	CDV^{+}	4	10.0	m	German Shepherd	n.d.
**39**	CDV^{+}	4	8.0	f	Retriever	n.d.
**40**	CDV^{+}	4	7.0	m	Mixed breed	E
**41**	CDV^{+}	4	84.0	m	Miniature Pinscher	E
**42**	CDV^{+}	4	12.0	f	Mixed breed	E
**43**	CDV^{+}	4	12.0	sf	Mixed breed	S
**44**	CDV^{+}	4	8.0	f	n.d.	E

Groups: 1 = non-infected controls; 2 = acute CDV infection; 3 = subacute CDV infection; 4 = subacute-chronic CDV infection; f = female; sf = spayed female; m = male; n.d. = no data; E = euthanasia; S = spontaneous death.

Supplementary Table S4. Anamnestic details of dogs used for RT-qPCR and RNA-Seq analysis

Dog No	CDV status	Group	Age [months]	Sex	Breed	Mode of death	RNA-Seq
**1**	non-infected	1	83	m	Dalmatian	E	X
**2**	non-infected	1	134	sf	Mixed breed	S	X
**3**	non-infected	1	n.d.	n.d.	n.d.	E	X
**4**	non-infected	1	178	f	Mixed breed	E	
**5**	non-infected	1	n.d.	n.d.	n.d.	E	
**6**	non-infected	1	2	f	Greater Swiss Mountain Dog	E	X
**7**	CDV^+	2	5.5	m	Old English Sheepdog	E	
**8**	CDV^+	2	n.d.	f	n.d.	n.d.	
**9**	CDV^+	2	3.0	f	Mixed breed	E	X
**10**	CDV^+	2	6.0	f	Mixed breed	S	X
**11**	CDV^+	2	n.d.	f	Mixed breed	n.d.	
**12**	CDV^+	2	3.5	f	Jack Russell Terrier	E	
**13**	CDV^+	2	3.0	f	English Bulldog	E	X
**14**	CDV^+	2	2.0	f	Mixed breed	E	X
**15**	CDV^+	2	6.0	f	n.d.	E	X

Groups: 1 = non-infected controls; 2 = acute CDV infection; f = female; sf = spayed female; m = male; n.d. = no data; E = euthanasia; S = spontaneous death; X = used for RNA-Seq analysis (in addition to RT-qPCR).
